# Case report: Concomitant *EGFR* mutation and *ALK* rearrangement in non-small cell lung cancer

**DOI:** 10.3389/fphar.2023.1167959

**Published:** 2023-08-29

**Authors:** Haoyue Hu, Songtao Tan, Meng Xie, Peng Guo, Qiang Yu, Juan Xiao, Kangrui Zhao, Qiong Liao, Yi Wang

**Affiliations:** ^1^ Department of Radiation Oncology, Sichuan Clinical Research Center for Cancer, Sichuan Cancer Hospital and Institute, Sichuan Cancer Center, Affiliated Cancer Hospital of University of Electronic Science and Technology of China, Chengdu, China; ^2^ West China School of Medicine, Sichuan University, Chengdu, China; ^3^ Department of Cleft Lip and Palate, Plastic Surgery Hospital, Chinese Academy of Medical Sciences and Peking Union Medical College, Shijingshan, Beijing, China; ^4^ Department of Pharmacy, Southwest Medical University, Luzhou, China; ^5^ Department of Pathology, Sichuan Clinical Research Center for Cancer, Sichuan Cancer Hospital and Institute, Sichuan Cancer Center, Affiliated Cancer Hospital of University of Electronic Science and Technology of China, Chengdu, China; ^6^ Dazhou Quxian People’s Hospital, Dazhou, China

**Keywords:** ALK rearrangement, EGFR mutation, lung adenocarcinoma, targeted therapy, case report

## Abstract

In non-small cell lung cancer (NSCLC), two key genetic alterations, epidermal growth factor receptor (*EGFR*) mutations and anaplastic lymphoma kinase (*ALK*) rearrangements, are commonly believed to be mutually exclusive. Studies have reported that concurrent *EGFR/ALK* co-mutation in non-small cell lung cancer patients is rare, with a prevalence ranging from 0.1% to 1.6%. However, the clinical and pathological characteristics of these patients are not well-defined, and the optimal treatment approach for such cases remains controversial. In this report, we present a case of stage IV lung adenocarcinoma with both epidermal growth factor receptor and anaplastic lymphoma kinase mutations, along with high PD-L1 expression. The patient initially received treatment with epidermal growth factor receptor tyrosine kinase inhibitors (TKIs), but the disease progressed. However, following a switch to *ALK*-TKI therapy and local radiotherapy, the lesion showed regression. Our report also provides a comprehensive summary of the clinical and pathological features, as well as treatment strategies, for non-small cell lung cancer patients with concurrent epidermal growth factor receptor mutation and anaplastic lymphoma kinase rearrangement.

## Introduction

Non-small cell lung cancer (NSCLC) is the most common type of lung cancer, accounting for approximately 80%–85% of cases (Zhou et al., 2021). In recent years, molecular genetics research on lung cancer has made remarkable progress, and the treatment of NSCLC has entered the era of targeted therapy. The most common driver gene mutation in NSCLC is epidermal growth factor receptor (*EGFR*), which is found in 45% of Asian patients and 20% of Caucasian patients with adenocarcinoma histology (Kim E. et al., 2021a; Kim ES. et al., 2021b). In individuals with sensitizing *EGFR* mutations, *EGFR*-tyrosine kinase inhibitors (TKIs) are suggested as first-line treatment. Anaplastic lymphoma kinase (*ALK*) rearrangement is less common than *EGFR* mutation, occurring in approximately 5% of NSCLC patients (Jahanzeb et al., 2020). *ALK*-TKIs are indicated as first-line treatment for individuals with *ALK* rearrangement. Early studies showed that *ALK* positivity and *EGFR* mutation are mutually exclusive and cannot coexist. However, recent studies have shown some cases or studies with coexistence of *EGFR* and *ALK* rearrangement, although this proportion of NSCLC is rare. Despite this, little is known about the molecular biology of these two oncogenes or the effect of *EGFR*-TKIs or *ALK* inhibitors in concomitant NSCLC patients. In this report, we present a case of NSCLC in a patient who harbored simultaneous *EGFR* mutation, *ALK* rearrangement, and high expression of PD-L1.

## Case presentation

**FIGURE 1:**
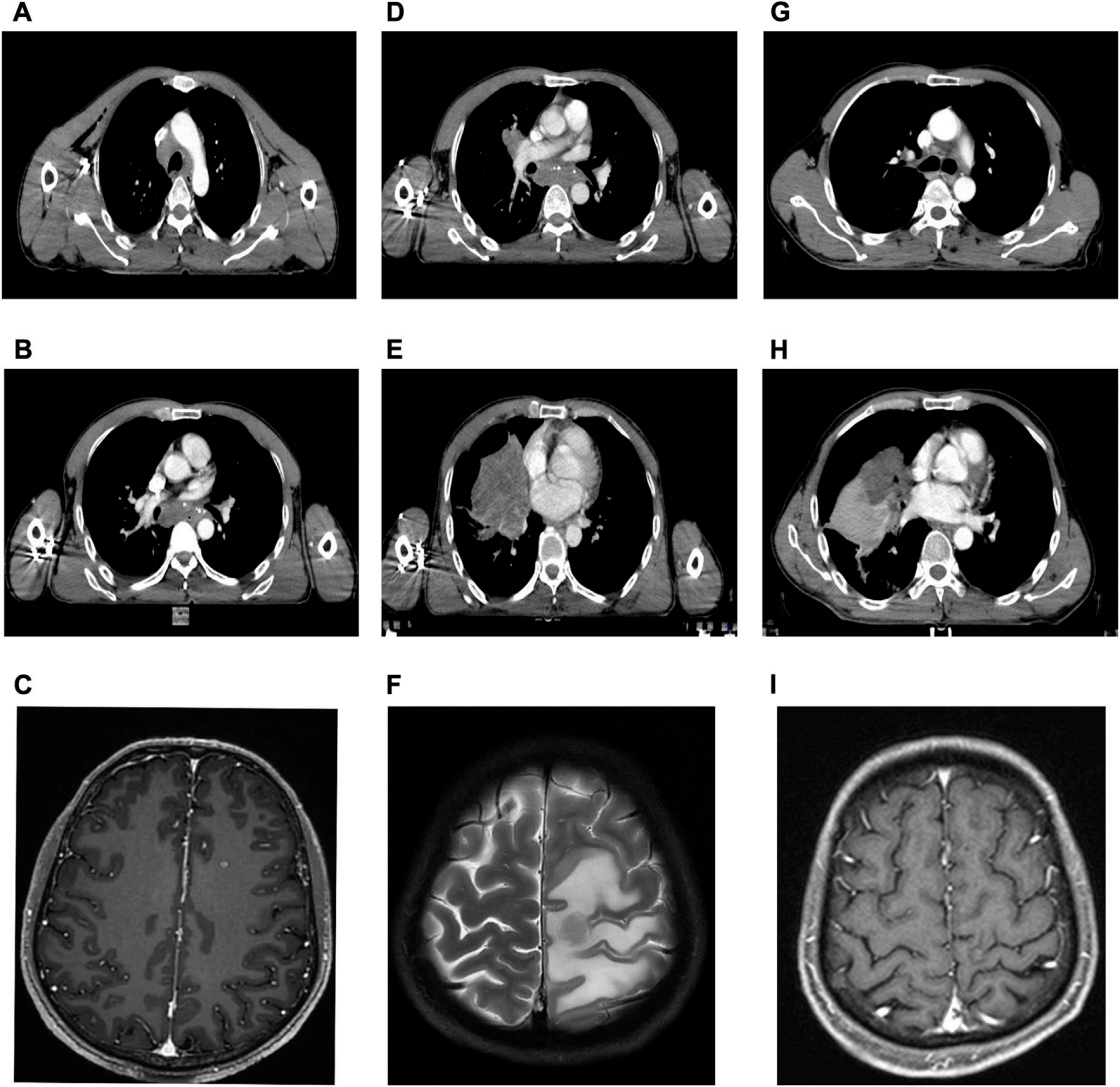
Serial CT and MRI of the case. **(A,B)** Baseline CT at diagnosis of the lung. **(C)** Baseline CT at diagnosis of the brain metastases. **(D,E)** CT after 3 months of osimertinib therapy with progressive disease of the lung. **(F)** MRI after 3 months of osimertinib therapy with progressive disease of the brain metastases with encephaledema. **(G,H)** CT after 3 months of alectinib therapy with a partial response of the lung. **(I)** MRI after 3 months of alectinib therapy and radiotherapy with a partial response of the brain metastases.

**FIGURE 2:**
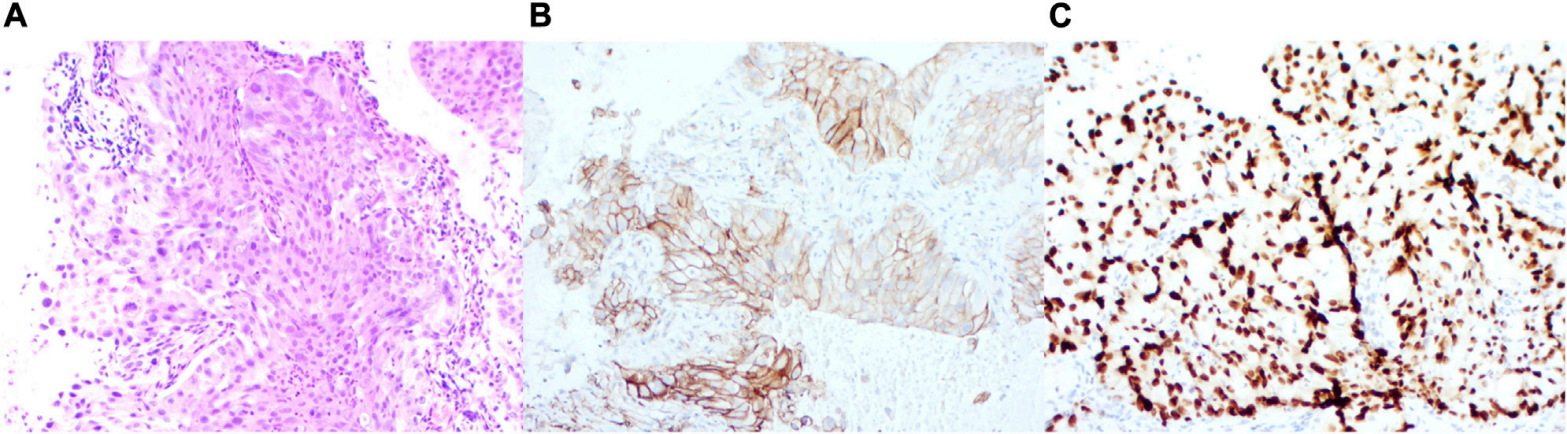
Immunohistochemical staining features. **(A)** Immunohistochemistry of the lung. **(B)** Expression patterns of PD-L1. **(C)** Expression patterns of TTF-1. Immunohistochemistry of *ALK* rearrangement.

In February 2022, a 54-year-old male heavy smoker presented to our hospital with persistent coughing and sputum production. A chest computed tomography (CT) scan revealed a mass measuring 4.2 cm × 3.3 cm in the middle and lower lobe of the right lung (Figures 1A, B). The scan also showed multiple enlarged lymph nodes in the bilateral supraclavicular fossa, mediastinum, and right hilar region. Further brain magnetic resonance imaging (MRI) and positron emission computed tomography (PET) scans indicated the presence of multiple metastases in the brain and ribs (Figure 1C). A transthoracic needle biopsy of the right lung mass revealed non-small cell carcinoma-favor adenocarcinoma (Figure 2). Based on these findings, the patient was diagnosed with stage IVB right middle and lower lobe adenocarcinoma, T4N3M1c.

**FIGURE 3:**
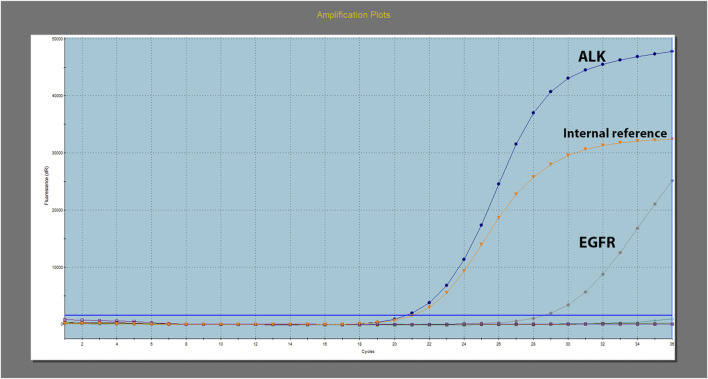
*EGFR* and *ALK* testing was performed by real-time polymerase chain reaction (PCR).

Further molecular screening using real-time polymerase chain reaction (PCR) revealed the presence of *EGFR* 19 exon deletion and echinoderm microtubule-associated protein-like 4-anaplastic lymphoma kinase (*EML4-ALK*) fusion (Figure 3). The test was conducted using the Human *EGFR/ALK/ROS1* Gene Mutation Combination Test Kit (Fluorescent PCR method) by Xiamen Aide, and the detection instrument used was Agilent MX3000P. Additionally, *EML4-ALK* was retested using immunohistochemistry, which showed strong positive expression of PD-L1 (>90%) (Figure 2).

Due to economic constraints, combined treatment with *EGFR*-TKIs and *ALK*-TKIs was not pursued. Instead, the patient began a daily dose of 80 mg of osimertinib (*EGFR*-TKI) in March 2022. However, after 2 months, a follow-up chest CT scan showed that the irregular mass in the right lung had increased in size compared to previous measurements. Multiple lymph node metastases had also grown larger, measuring up to 3 cm × 2 cm (Figure 1D-e). A subsequent brain MRI revealed multiple scattered nodules in both brain hemispheres, which had increased in both size and quantity since the last examination (Figure 1F). The therapeutic response was determined to be progressive disease (PD), resulting in an initial progression-free survival (PFS) of 2 months.

Subsequently, the patient received twice daily administration of 600 mg alectinib (*ALK*-TKI) due to the presence of the *EML4-ALK* fusion gene. Stereotactic body radiotherapy (SBRT) was employed eight times, with a cumulative dose of 5 Gy/F, to address brain metastases. Additionally, bevacizumab (200 mg/q3w) was administered to mitigate brain edema. After 2 weeks of treatment, follow-up chest CT and brain MRI scans showed a noticeable reduction in the size of the mass in the right lung, enlarged lymph nodes, and brain metastasis (Figure 1H-j). Furthermore, subsequent therapy with alectinib resulted in a partial response (PR) thus far. The sum of the maximum diameter of the tumor target lesions was reduced by ≥ 30% and maintained for at least 4 weeks. Alectinib therapy will continue, and the decision regarding chest radiotherapy will be based on subsequent examinations.

## Discussion

In this report, we describe a patient with a rare molecular subtype of NSCLC harboring concurrent *EGFR* mutations and *ALK* rearrangement. To our knowledge, there is little information available on the therapeutic efficacy of *EGFR*-TKIs and *ALK*-TKIs for these patients, with the exception of a small number of studies that reveal inconsistent findings regarding the response to the two TKIs (Won et al., 2015; Zhou et al., 2015; Lo Russo et al., 2017; Schmid et al., 2017). Additionally, choosing the optimum targeted medication in this circumstance is challenging, since there is no clinical guideline that specifies the order in which *EGFR*-TKIs and *ALK*-TKIs should be used.

To date, ≥107 patients with a concurrent *EGFR* mutation and *ALK* rearrangement have been documented, and their clinical features and treatment outcomes are listed in Table 1 ((5-7), ()). Inferring from the data in Table 1, we discovered that 86 patients had received an *EGFR*-TKI, 2 had achieved complete response (CR), 25 partial response (PR), 19 objective response (OR, OR = CR + PR), 15 stable disease (SD), and 25 progressive disease (PD). The objective response rate (ORR) was 53.5% (46 of 86), while the disease control rate (DCR) was 70.9% (61 of 86). In addition, *ALK*-TKI was administered to 50 patients, of whom 1 experienced CR, 22 PR, 8 OR, 7 SD, and 12 PD. The ORR was 62.0% (31 of 50), and the DCR was 76% (38 of 50). Compared with previous reports (Zhao et al., 2015), the most recent data, which encompassed a large number of cases, revealed a decrease in the overall response rate (ORR) of *EGFR*-TKIs, while patients treated with *ALK*-TKIs had an increased ORR. These results align with our own observations. Furthermore, the data showed that cases with *EGFR* and *ALK* concomitant mutations were more frequently reported in East Asian populations, which was consistent with the higher *EGFR* mutation rate in East Asian patients than in Caucasians (Paez et al., 2004).

**TABLE 1:** A total of 107 patients with a concurrent *EGFR* mutation and *ALK* rearrangement have been documented, and their clinical features and treatment outcomes are listed.

Investigator	Time	Patients (n)	Age (years)	Sex	Race	Smoking history	Histologic type	TNM stage	Method	First EGFR-TKI	Response	First ALK-TKI	Response
Kuo et al., 2010	2010	1	72	F	Asian	No	AC	IV	Single	Gefitinib	PR PFS, 7.7 m	ND	-
Tiseo et al., 2011	2010	1	48	M	White	No	SC/AC	NR	Single	Erlotinib	PD	ND	-
Popat et al., 2011	2011	1	65	F	White	No	AC	IIIA	Single	Erlotinib	CR PFS, 25 m	ND	-
Tanaka et al., 2012	2012	1	39	M	Asian	Yes	AC	IV	Single	Erlotinib	PD	ND	-
Santelmo et al., 2013	2013	1	52	F	White	Yes	AC	IIIA	Single	Gefitinib	SD PFS, 7 m	ND	-
Chen et al. (2013)	2013	1	56	M	Asian	Yes	AC	IV	Single	Erlotinib	SD PFS, 8 m	Crizotinib	CR PFS, >22 m
Miyanaga et al., 2013	2013	1	55	F	Asian	No	AC	IV	Single	Gefitinib	PD	NR	SD PFS, 4 m
Zhao et al. (2015)	2014	1	48	F	Asian	No	AC	IV	Single	Erlotinib	SD PFS, 5.3 m	Crizotinib	SD PFS, 3.5 m
Zhou et al. (2015)	2014	1	47	F	Asian	No	AC	IV	Single	Gefitinib	PD	ND	-
Chiari et al. (2014)	2014	1	67	F	White	No	AC	IV	Single	Gefitinib	PR PFS, 24 m	Crizotinib	PR PFS, 25 m
Yang et al., 2014	2014	13	Median age, 59	M, 5 F, 8	Asian	No, 12 Yes, 1	AC	IIIA, 2 IV, 11	Single	Gefitinib, 3 Erlotinib, 5 Afatinib, 2	3 PR 4 PR, 1 PD 1 PR, 1 SD Median PFS 11.2 m	Crizotinib, 4	2 PR, 1 SD, 1 PD
Won et al. (2015)	2014	14	Median age, 55.5	M, 6 F, 8	Asian	No, 12 Yes, 2	13 AC 1 NR	IA, 1 IB, 1 IV, 11	Single	Gefitinib, 3	1 SD, 2 PD	Crizotinib, 8	7 PR, 1SD
Xu et al., 2015	2015	1	71	F	Asian	No	AC	IV	Single	Gefitinib	SD PFS, 8 m	ND	-
Ulivi et al., 2016	2015	6	Median age, 61	M, 1 F, 5	White	No, 3 Yes, 3	AC	NR	Single	Gefitinib, 5 Erlotinib, 1	1 CR, 2 PR, 2 PD 1 PR	ND	-
Schmid et al. (2017)	2016	5	Median age, 60	M, 2 F, 3	White	No, 3 Yes, 2	AC	IVA, 1 IVB, 4	Single,4 Dual, 1	Afatinib, \ Osimertinib, \ Erlotinib, \ (n = 4)	3 ORR, 1 SD Median PFS, 5.8 m	Alectinib, 1 Crizotinib, 2	1 PR 1 PR, 1 SD Median PFS, 5.7 m
Mao and Wu, (2017)	2017	10	Median age, 55.5	M, 4 F, 6	Asian	No, 9 Yes, 1	AC	IV	Single	Erlotinib, \ Gefitinib, \ Icotinib, \ (n = 10)	5 PR, 3 SD, 2 PD	Crizotinib, 5	3 PR, 1 SD, 1 PD
Shin et al., 2019	2019	3	Median age, 57	M, 1 F, 2	Asian	No	AC	IV	Single	Gefitinib, 1 Osimertinib, 2	1 PR 1 PR; 1 PD	Crizotinib, 2	2 PD
Lu et al., 2021	2021	1	72	M	Asian	No	AC	IIB	Single	Afatinib	PR PFS, 25 m	ND	-

UK: unknown.

NA: not applicable.

ND: not done.

*EGFR* mutation and *ALK* rearrangement were previously believed to be mutually exclusive (Horn and Pao, 2009; Shaw et al., 2009) and to be mutual causes of resistance to *EGFR*-TKIs or *ALK*-TKIs (Sasaki et al., 2011; Liang et al., 2016). In contrast, studies have shown that it is possible for patients harboring concurrent *EGFR* and *ALK* mutations to respond to both *EGFR*-TKIs and *ALK*-TKIs (Chen et al., 2013; Chiari et al., 2014; Schmid et al., 2017). This implied that these individuals may not be resistant to both *EGFR*-TKIs and *ALK*-TKIs but could instead obtain different responses with one of these medications. The degree of the relevant gene modifications may determine which medication is more effective if the tumor is caused by two different driver genes (Zhao et al., 2015). In addition, as the degree of signaling pathway activation is correlated with the level of phosphorylation of downstream proteins, the detection of the abundance of *EGFR* mutations and *ALK* rearrangements and the levels of phosphorylation of downstream proteins is essential to optimize the selection of TKIs in clinical practice (Lou et al., 2016).

Furthermore, it is also important to determine when *EGFR/ALK* coalterations occur and the subtype of *ALK* rearrangement. An earlier study discovered that patients with *EML4-ALK/EGFR* coalterations frequently had a significantly shorter median PFS than patients with non-*EML4-ALK/EGFR* coalterations after receiving *EGFR*-TKI therapy (Liu et al., 2019). According to the author, non-*EML4-ALK* coalterations may be an acquired resistance mechanism brought on by *EGFR*-TKIs, while *EML4-ALK* coalterations were likely to be a *de novo* change. Additionally, there are also two possibilities for the case where both genetic alterations exist from the beginning of tumor proliferation. The two biomolecular alterations may coexist in different cellular clones, which represents the expression of the heterogeneity of tumors or the same tumor cell. All mechanisms are possible and may emerge in the same patient at different times over the course of the illness (Lo Russo et al., 2017). Since the two alterations may coexist *ab initio*, the combination of *EGFR*-TKIs and *ALK*-TKIs may exert satisfactory efficacy, and further study is needed (Noronha et al., 2022).

In our case, due to economic constraints, the patient did not opt for combination therapy with *EGFR*-TKI and *ALK*-TKI. As there is no consensus on treatment for patients with *EGFR/ALK* coalterations, we selected *EGFR*-TKI as the first-line treatment for the patient, as recommended by some studies. The efficacy of alectinib and lack of efficacy of osimertinib may be due to the higher abundance of *ALK* rearrangement and relatively higher activation of *ALK* than the *EGFR* signaling pathway (Lou et al., 2016). However, a limitation of this case report is the lack of measurement of mutation abundance and downstream pathway activation. Additionally, the overexpression of PD-L1 (>90%) in our patient may potentially negatively impact the efficacy of TKIs and further complicate treatment options (He et al., 2022). Patients with *ALK* mutations have a significantly higher incidence of high PD-L1 expression (≥50%). Previous research has shown that NSCLCs with *ALK* rearrangements have limited objective response rates and a short median progression-free survival (PFS) when treated with PD-1/PD-L1 inhibitors. Moreover, PD-L1 expression was not found to be a crucial biomarker for immune checkpoint inhibitor (ICI) therapy in patients with genetic mutations (Kris et al., 2011; Barlesi et al., 2013). For patients harboring dual mutations and exhibiting high PD-L1 expression, further clinical trials are imperative to validate the efficacy of combining immunosuppressive drugs and assess the manageability of potential side effects.

As part of the initial diagnosis, *EGFR, ALK, KRAS*, and other gene mutations must be detected before treatment. Oncologists must take into account the presence of dual or multiple oncogenes when selecting the most suitable therapeutic strategies, which may include combination or sequential treatment methods. However, more research is required to gain a deeper understanding of therapeutic approaches in patients with both *EGFR* mutation and *ALK* rearrangement.

## Conclusion

In conclusion, we presented a case of a patient with both *EGFR* mutation and *ALK* rearrangement, who experienced disease progression (PD) after osimertinib treatment. Subsequent therapy with alectinib resulted in a partial response (PR) so far. Recent data, including more cases, have suggested that *ALK*-TKIs may have better efficacy than *EGFR*-TKIs, which is consistent with our case. Moreover, it is essential to evaluate the abundance of gene mutations and levels of downstream protein phosphorylation to optimize treatment outcomes. Further research is required to gain a better understanding of the molecular mechanisms underlying responsiveness and resistance to *EGFR*-TKIs and *ALK*-TKIs in this specific subgroup of patients with coalterations. Additionally, exploring potential combination or sequential therapy strategies is necessary.

## Data Availability

The original contributions presented in the study are included in the article/supplementary material, further inquiries can be directed to the corresponding author.
